# Supplementary material for: HIVprotI: an integrated web based platform for prediction and design of HIV proteins inhibitors
Source: J Cheminform. 2018 Mar 9;10:12. doi: 10.1186/s13321-018-0266-y (PMC5845081; doi:10.1186/s13321-018-0266-y)
Supplement: Supplementary file 2 — Additional file 2. Source code of HIVProtI web server. [file 13321_2018_266_MOESM2_ESM.zip › HIVprotI_Source-code/hivprotI/weka-3-6-6/doc/deprecated-list.html]

Deprecated List


---


|  |  |  |
| --- | --- | --- |
| **Overview** \| Package \| Class \| **Tree** \| **Deprecated** \| **Index** \| **Help** \| **Weka's home** | |  |
| PREV   NEXT | **FRAMES**    **NO FRAMES**     **All Classes** |  |


---


## **Deprecated API**


---

**Contents**

- Deprecated Classes- Deprecated Methods- Deprecated Constructors

**Deprecated Classes**

weka.core.Matrix
  
          *Use `weka.core.matrix.Matrix` instead - only for
backwards compatibility.*

**Deprecated Methods**

weka.clusterers.Cobweb.addInstance(Instance)
  
          *updateClusterer(Instance) should be used instead*

weka.core.Instances.readInstance(Reader)
  
          *instead of using this method in conjunction with the
`readInstance(Reader)` method, one should use the
`ArffLoader` or `DataSource` class instead.*

**Deprecated Constructors**

weka.core.Instances(Reader, int)
  
          *instead of using this method in conjunction with the
`readInstance(Reader)` method, one should use the
`ArffLoader` or `DataSource` class instead.*

weka.gui.PropertyDialog(PropertyEditor, int, int)
  
          *instead of this constructor, one should use the constructors
with an explicit owner (either derived from
`java.awt.Dialog` or from
`java.awt.Frame`) or, if none available,
using `(Frame) null` as owner.*

---


|  |  |  |
| --- | --- | --- |
| **Overview** \| Package \| Class \| **Tree** \| **Deprecated** \| **Index** \| **Help** \| **Weka's home** | |  |
| PREV   NEXT | **FRAMES**    **NO FRAMES**     **All Classes** |  |


---
